# Supplementary material for: Orbital-selective Dirac fermions and extremely flat bands in frustrated kagome-lattice metal CoSn
Source: Nat Commun. 2020 Aug 10;11:4002. doi: 10.1038/s41467-020-17462-4 (PMC7417585; doi:10.1038/s41467-020-17462-4)
Supplement: Supplementary file 1 — Supplementary Information [file 41467_2020_17462_MOESM1_ESM.pdf]

Supplementary Information:

Orbital-selective Dirac fermions and extremely flat bands in frustrated kagome-lattice metal CoSn

Liu et al.

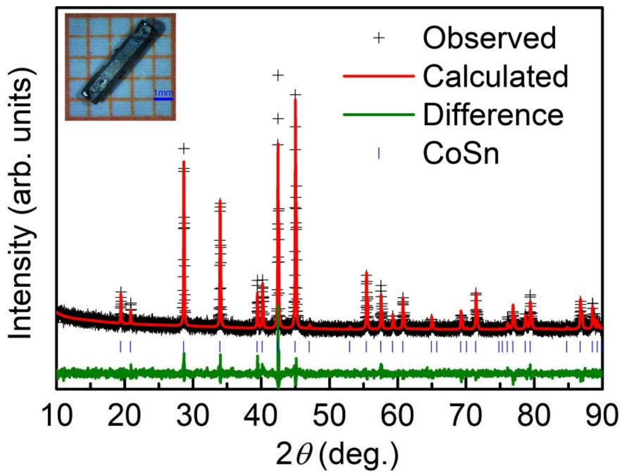

Supplementary Figure 1. Powder XRD pattern and Rietveld fit of ground CoSn single crystals. Inset: photo of CoSn single crystal on 1-mm grid paper.

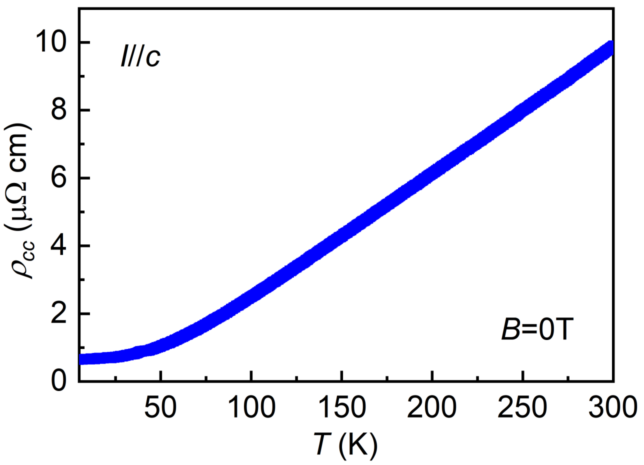

Supplementary Figure 2. Temperature dependence of resistivity $\rho_{cc}(T)$ of a CoSn single crystal at zero field.

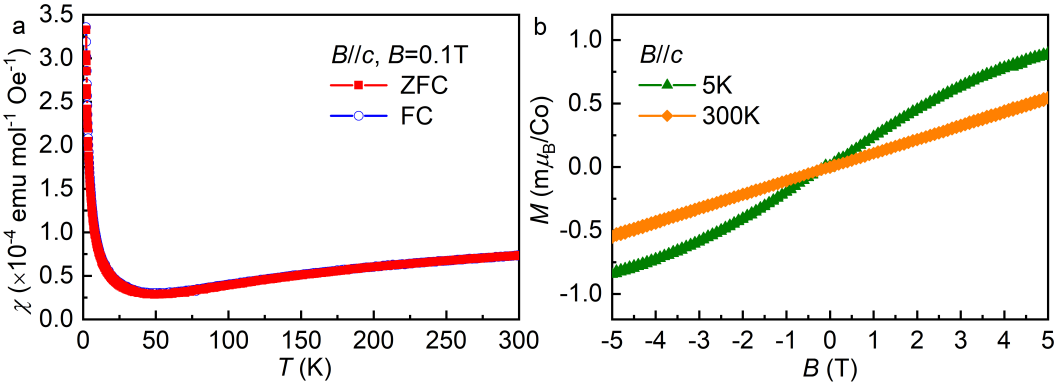

Supplementary Figure 3. a Temperature dependence of magnetic susceptibility $\chi(T)$ with zero field cooling and field cooling modes at $B = 0.1$ T for $B \| c$. **b** Field dependence of magnetization $M(B)$ at 5 K and 300 K.

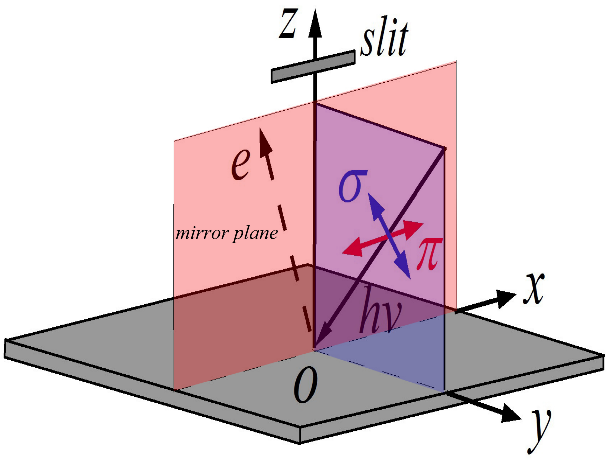

Supplementary Figure 4. Sketch of ARPES experimental setup. The electric field in π geometry is primarily along the x direction, and in σ geometry along y direction, and contains a z-component.

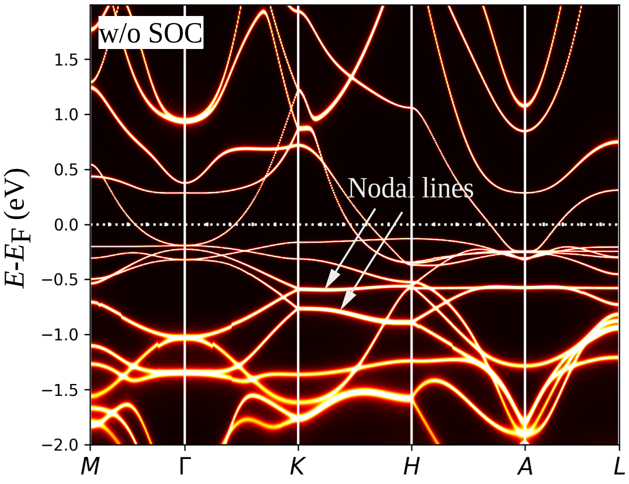

Supplementary Figure 5. DFT+DMFT calculation without SOC along $M - \Gamma - K - H - A - L$ lines.

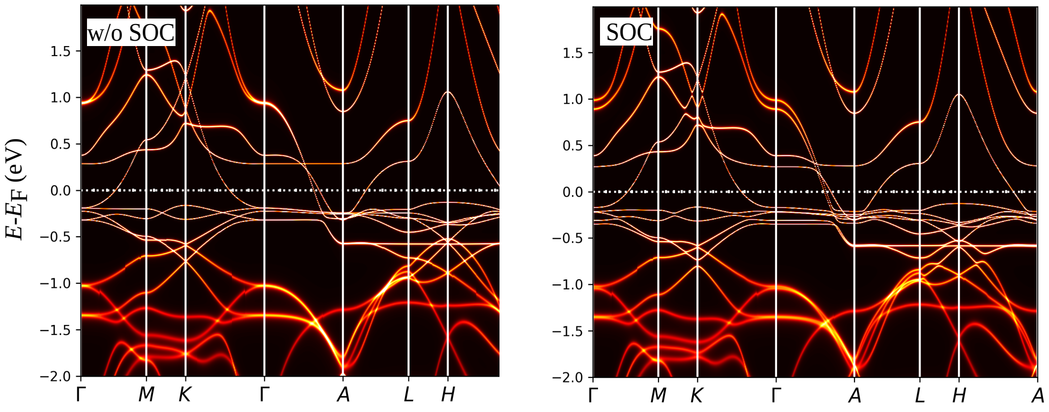

Supplementary Figure 6. DFT+DMFT calculation without and with SOC, respectively.

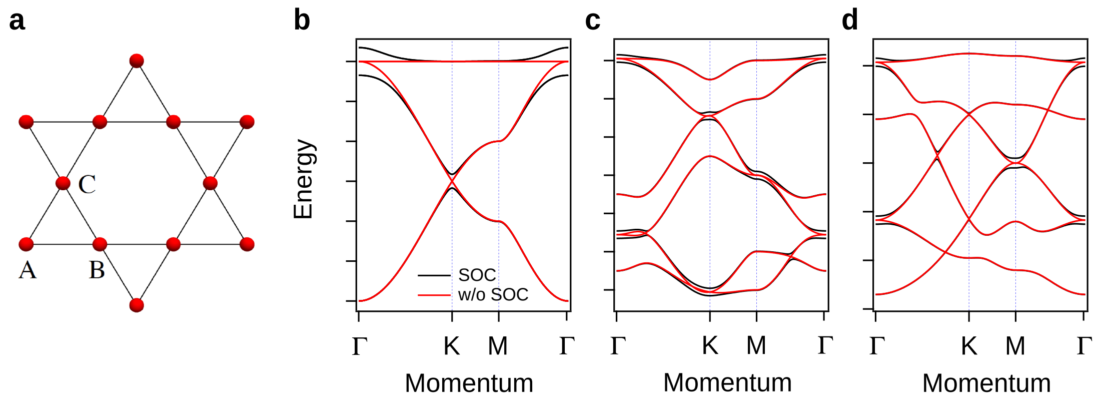

Supplementary Figure 7. **a** 2D kagome lattice with atoms in each unit cell labeled by A, B, and C. **b** The nearest neighbor single band calculation without SOC (red) and with (black) Kane-Mele SOC $\lambda = 0.1$ [1]. **c** Band structures of the two-orbital model with $t_\pi = 1.0, t_\delta = 1.0, t'_\pi = -0.5$, and $\lambda = 0.1$. **d** Band structures of the two-orbital model with $t_\pi = 1.0, t_\delta = -0.3, t'_\pi = 0.3, t'_\delta = -0.1$, and $\lambda = 0.1$.

	E_D (eV)		V_D (eV·Å) ($\Gamma - K$)		E_Δ (eV) (K point)		$W_{\Gamma-K}$ (eV)		$W_{\Gamma-M}$ (eV)	
Orbitals	d_{xy}/d_{x2-y2}	d_{xz}/d_{yz}	d_{xy}/d_{x2-y2}	d_{xz}/d_{yz}	d_{xy}/d_{x2-y2}	d_{xz}/d_{yz}	d_{xy}/d_{x2-y2}	d_{xz}/d_{yz}	d_{xy}/d_{x2-y2}	d_{xz}/d_{yz}
DFT	-0.99	-0.96	1.29±0.02	1.01±0.02	0.85	0.40	0.2	0.18	0.1	0.04
DFT+DMFT	-0.77	-0.59	0.91±0.02	0.63±0.02	0.60	0.28	0.08	0.12	0.1	0.02
ARPES	-0.6±0.05	-0.6±0.05	0.96±0.02	0.76±0.02	0.53±0.05	0.24±0.05	0.2	0.12	0.1	0.02
DFT/ARPES	-	-	1.34±0.06	1.33±0.06	1.62±0.2	1.74±0.4	1	1.5	1	2

Supplementary Table 1. The energy positions (E_D) and the velocity (V_D) of Dirac cones, the relative energy shift between Dirac points and flat bands (E_Δ), and the bandwidth of flat bands ($W_{\Gamma-K}$ and $W_{\Gamma-M}$) estimated from DFT, DFT+DMFT, and ARPES results.

Supplementary Note 1. Sample growth and characterizations. High quality single crystals of CoSn were synthesized by the Sn flux method. The starting elements of Co (99.99 %), Sn (99.99 %) were put into an alumina crucible, with a molar ratio of Co : Sn = 3 : 20. The mixture was sealed in a quartz ampoule under partial argon atmosphere and heated up to 1173 K, then cooled down to 873 K with 2 K/h. The CoSn single crystals were separated from the Sn flux by using centrifuge. The CoSn crystals are characterized by powder x-ray diffraction (XRD), resistivity and magnetization measurements (Supplementary Figs. 1-3). As shown in Supplementary Fig. 1, the XRD pattern of ground crystals can be well fitted by using the reported structure of CoSn (space group P6/mmm, No. 191) [2]. The inset shows a typical CoSn crystal and the long axis of rod-like crystal is the crystallographic *c* axis. Supplementary Fig. 2 shows the temperature dependence of *c*-axial resistivity $\rho_{cc}(T)$. The decrease of $\rho_{cc}(T)$ with decreasing temperature clearly indicates the metallic behavior of CoSn. Supplementary Fig. 3a shows the temperature dependent magnetic susceptibility $\chi(T)$ with zero field cooling and field cooling modes at $B = 0.1$ T for $B \| c$. Both ZFC and FC $\chi(T)$ curves overlap each other very well and exhibit weak temperature independence above 50 K with small and positive values, which supports Pauli paramagnetic behavior of CoSn. On the other hand, there are small Curie tails observed at low temperature, which could originate from trace amounts of magnetic impurities in CoSn. We attribute it extrinsic for similar results being reported and discussed in previous study [2]. For the field dependence of magnetization $M(B)$ (Supplementary Fig. 3b), the small and unsaturated $M(B)$ values up to 5 T (only about 0.9 mμ_B/Co at 2 K) further confirm the paramagnetic behavior of CoSn.

Supplementary Note 2. Angle-resolved photoemission spectroscopy experiments. Angle-resolved photoemission spectroscopy experiments (ARPES) measurements were performed at dreamline and 03U beamlines of Shanghai Synchrotron Radiation Facility (SSRF). Samples were cleaved *in situ*, yielding a flat mirror-like (001) surface. During measurements, the temperature was kept at $T = 20$ K. The pressure was maintained greater than 5×10^{11} Torr. ARPES experimental setup is shown in Supplementary Fig. 4. The intensity of each band is dominated by the matrix element which depends on symmetry of the band, the polarization of incident photons, and their relative angle. The linear polarization of the photons can be exploited in two different experimental geometries (π and σ). The high symmetry direction and the normal of the sample surface define a mirror plane. The π (σ) geometry refers to the electric fields of the incident photons within (normal to) the mirror plane, respectively. Assuming the final-state of the outgoing electron depicted by a plane wave, the wave function remains even with respect to the plane [3]. Thus the d_{x2-y2}, d_{xz}, and d_{z2} are of even symmetry, while the d_{xy} and d_{yz} are of odd symmetry with respect to the mirror plane. The orbitals with even (odd) symmetry with respect to the mirror plane are detected in π (σ) geometry.

Here, we note that our experimental setup for the σ geometry includes a polarization component along z. Thus the d_{xz}, d_{yz} and d_{z2} orbitals can be enhanced in σ geometry by the z-component light. Because the point group of CoSn is D_{6h}, $3d$ orbitals are divided into three groups: in-plane d_{xy}/d_{x2-y2}, out-of-plane d_{xz}/d_{yz}, and d_{z2}. The orbital combinations, e.g. d_{xy} (odd) and d_{x2-y2} (even) [d_{xz} (even) and d_{yz} (odd)], could be enhanced or suppressed individually, thus the Dirac cones and the flat bands features could be more prominent under certain conditions.

Supplementary Note 3. Calculations. Density functional theory (DFT) calculation was done in the non-magnetic phase whereas dynamical mean field theory (DFT+DMFT) calculation was done in the paramagnetic phase. For DFT calculations, we used the full-potential linear augmented plane wave method implemented in Wien2K [4] in conjunction with Perdew-Burke-Ernzerhof generalized gradient approximation [5] of the exchange correlation functional. DFT+DMFT was implemented on top of Wien2K and documented in Ref. [6]. In the DFT+DMFT calculations, the electronic charge was computed self-consistently on DFT+DMFT density matrix. The quantum impurity problem was solved by the continuous time quantum Monte Carlo (CTQMC) method [6–8], at a temperature of 116 K and with a Hubbard $U = 5.0$ eV and Hund's rule coupling $J = 0.8$ eV in the paramagnetic state. The same values as we used for many iron-based compounds [9–11]. The experimental crystal structure (space group $P6/mmm$, No. 191) of CoSn with lattice constants $a = b = 5.275$ Å and $c = 4.263$ Å was used in the calculations. In the DFT+DMFT calculation, the DFT solution is non-spin-polarized, the DMFT impurity problem was then constructed using both spins with rotational invariant Coulomb interactions, which allows the impurity atom to form a non-zero local fluctuating spin (the time average of the local fluctuating spin is zero, i.e., no net spin moment), thus forming paramagnetic state [7]. There is a negligible sign problem at the temperature of the calculation. We did not neglect anything intentionally in the procedure.

Results of DFT+DMFT calculations along the high-symmetry lines are shown in Supplementary Figs. 5, 6. Supplementary Fig. 5 shows DFT+DMFT calculated bands without spin-orbit coupling (SOC) along the $M-\Gamma-K-H-A-L$ directions, revealing two Dirac nodal lines along the $K-H$ direction, protected by the spatial-inversion symmetry and time-reversal symmetry, when SOC is ignored. Supplementary Fig. 6 shows DFT+DMFT calculated bands without and with SOC, respectively, along the $\Gamma-M-K-\Gamma-A-L-H-A$ directions. Supplementary Table1 shows the energy positions (E_D) and the velocity (V_D) of Dirac cones, the relative energy shift between Dirac points and flat bands (E_Δ), and the bandwidth of flat bands ($W_{\Gamma-K}$ and $W_{\Gamma-M}$) estimated from DFT, DFT+DMFT, and ARPES results.

Supplementary Note 4. Tight-binding calculation. In the CoSn crystal, the Co atoms form a kagome

lattice with Sn atoms locating at the kagome lattice hexagonal center. Based on the experimental and calculational findings, we propose that a two-orbital model can be used to describe the extremely flat band of d_{xz}/d_{yz} orbital. From DFT and DFT+DMFT calculations along k_z ($\Gamma - A$), we know that d_{xz}/d_{yz} bands are quite two-dimensional in the vicinity of E_F. Hence, we consider only the hopping integrals along the Co plane and ignore the interlayer coupling. Using the Slater-Koster integrals [12], the two orbital model with d_{xz}/d_{yz} character can be constructed as $H = H_{1N} + H_{2N} + H_{soc}$, where the H_{1N} and H_{2N} describe the nearest-neighbor and second nearest-neighbor hopping respectively. H_{soc} is the atomic SOC. Using the basis $(d_{xz,A}, d_{xz,B}, d_{xz,C}, d_{yz,A}, d_{yz,B}, d_{yz,C})^T$, each term can be written as

$$H_{1N} = \begin{bmatrix} 0 & 2t_\pi \cos\frac{k_1}{2} & \frac{t_\pi+3t_\delta}{2}\cos\frac{k_2}{2} & 0 & 0 & \frac{\sqrt{3}(t_\pi-t_\delta)}{2}\cos\frac{k_2}{2} \\ 2t_\pi \cos\frac{k_1}{2} & 0 & \frac{t_\pi+3t_\delta}{2}\cos\frac{k_3}{2} & 0 & 0 & -\frac{\sqrt{3}(t_\pi-t_\delta)}{2}\cos\frac{k_3}{2} \\ \frac{t_\pi+3t_\delta}{2}\cos\frac{k_2}{2} & \frac{t_\pi+3t_\delta}{2}\cos\frac{k_3}{2} & 0 & \frac{\sqrt{3}(t_\pi-t_\delta)}{2}\cos\frac{k_2}{2} & -\frac{\sqrt{3}(t_\pi-t_\delta)}{2}\cos\frac{k_3}{2} & \\ 0 & 0 & \frac{\sqrt{3}(t_\pi-t_\delta)}{2}\cos\frac{k_2}{2} & 0 & 2t_\delta \cos\frac{k_1}{2} & \frac{3t_\pi+t_\delta}{2}\cos\frac{k_2}{2} \\ 0 & 0 & -\frac{\sqrt{3}(t_\pi-t_\delta)}{2}\cos\frac{k_3}{2} & 2t_\delta \cos\frac{k_1}{2} & 0 & \frac{3t_\pi+t_\delta}{2}\cos\frac{k_3}{2} \\ \frac{\sqrt{3}(t_\pi-t_\delta)}{2}\cos\frac{k_2}{2} & -\frac{\sqrt{3}(t_\pi-t_\delta)}{2}\cos\frac{k_3}{2} & 0 & \frac{3t_\pi+t_\delta}{2}\cos\frac{k_2}{2} & \frac{3t_\pi+t_\delta}{2}\cos\frac{k_3}{2} & 0 \end{bmatrix}$$

$$H_{2N} = \begin{bmatrix} 0 & 2t'_\delta \cos k_4 & \frac{3t'_\pi+t'_\delta}{2}\cos k_5 & 0 & 0 & -\frac{\sqrt{3}(t'_\pi-t'_\delta)}{2}\cos k_5 \\ 2t'_\delta \cos k_4 & 0 & \frac{3t'_\pi+t'_\delta}{2}\cos k_6 & 0 & 0 & \frac{\sqrt{3}(t'_\pi-t'_\delta)}{2}\cos k_6 \\ \frac{3t'_\pi+t'_\delta}{2}k_5 & \frac{3t'_\pi+t'_\delta}{2}\cos k_6 & 0 & -\frac{\sqrt{3}(t'_\pi-t'_\delta)}{2}\cos k_5 & \frac{\sqrt{3}(t'_\pi-t'_\delta)}{2}\cos k_6 & \\ 0 & 0 & -\frac{\sqrt{3}(t'_\pi-t'_\delta)}{2}\cos k_5 & 0 & 2t'_\pi \cos k_4 & \frac{t'_\pi+3t'_\delta}{2}\cos k_5 \\ 0 & 0 & \frac{\sqrt{3}(t'_\pi-t'_\delta)}{2}\cos k_6 & 2t'_\pi \cos k_4 & 0 & \frac{t'_\pi+3t'_\delta}{2}\cos k_6 \\ -\frac{\sqrt{3}(t'_\pi-t'_\delta)}{2}\cos k_5 & \frac{\sqrt{3}(t'_\pi-t'_\delta)}{2}\cos k_6 & 0 & \frac{t'_\pi+3t'_\delta}{2}\cos k_5 & \frac{t'_\pi+3t'_\delta}{2}\cos k_6 & 0 \end{bmatrix}$$

$$H_{soc} = \begin{bmatrix} 0 & 0 & 0 & -i\lambda & 0 & 0 \\ 0 & 0 & 0 & 0 & -i\lambda & 0 \\ 0 & 0 & 0 & 0 & 0 & -i\lambda \\ i\lambda & 0 & 0 & 0 & 0 & 0 \\ 0 & i\lambda & 0 & 0 & 0 & 0 \\ 0 & 0 & i\lambda & 0 & 0 & 0 \end{bmatrix}$$

with $k_1 = k_x, k_2 = \frac{k_x}{2} + \frac{\sqrt{3}k_y}{2}, k_3 = k_2 - k_1, k_4 = k_2 - \frac{k_1}{2}, k_5 = k_1 - \frac{k_2}{2}, k_6 = \frac{k_1+k_2}{2}$, where A, B, and C is the site label in each unit cell, as shown in Supplementary Fig. 7a. In this model, we can define a global energy scale $t = 1.0$. By tuning the hopping parameter to a flat band can be obtained as shown in Supplementary Figs. 7b-d. There is also a quadratic band touching at the Γ point, which can be further gapped out by inducing SOC. Note that, in contrast to nearest neighbor single band model in kagome lattice, the flat band in the two-orbital model needs the cooperation of different neighbor's hopping integrals. The two-orbital

model will reduce to single-orbital model when $t_\pi = t_\delta$, and $t'_\pi = t'_\delta$.

Supplementary references

[1] Yin, J. *et al.* Giant and anisotropic many-body spin orbit tunability in a strongly correlated kagome magnet. *Nature* **562**, 91–95 (2018).

[2] Allred, J. M., Jia, S., Bremholm, M. & Cava, R. J. Ordered CoSn-type ternary phases in $Co_3Sn_{3-x}Ge_x$. *J. Alloys Compounds* **539**, 137–143 (2012).

[3] Damascelli, A., Hussain, Z. & Shen, Z.-X. Angle-resolved photoemission studies of the cuprate superconductors. *Rev. Mod. Phys.* **75**, 473–541 (2003).

[4] Blaha, P., Schwarz, K., Madsen, G. K., Kvasnicka, D. & Luitz, J. Wien2k. An augmented plane wave+ local orbitals program for calculating crystal properties (2001).

[5] Perdew, J. P., Burke, K. & Ernzerhof, M. Generalized gradient approximation made simple. *Phys. Rev. Lett.* **77**, 3865–3868 (1996).

[6] Haule, K. Quantum Monte Carlo impurity solver for cluster dynamical mean-field theory and electronic structure calculations with adjustable cluster base. *Phys. Rev. B* **75**, 155113 (2007).

[7] Haule, K., Yee, C.-H. & Kim, K. Dynamical mean-field theory within the full-potential methods: Electronic structure of $CeIrIn_5$, $CeCoIn_5$, and $CeRhIn_5$. *Phys. Rev. B* **81**, 195107 (2010).

[8] Werner, P., Comanac, A., de' Medici, L., Troyer, M. & Millis, A. J. Continuous-time solver for quantum impurity models. *Phys. Rev. Lett.* **97**, 076405 (2006).

[9] Yin, Z. P., Haule, K. & Kotliar, G. Spin dynamics and orbital-antiphase pairing symmetry in iron-based superconductors. *Nat. Phys.* **10**, 845 (2014).

[10] Yin, Z. P., Haule, K. & Kotliar, G. Magnetism and charge dynamics in iron pnictides. *Nat. Phys.* **7**, 294 (2011).

[11] Yin, Z. P., Haule, K. & Kotliar, G. Kinetic frustration and the nature of the magnetic and paramagnetic states in iron pnictides and iron chalcogenides. *Nat. Mater.* **10**, 932 (2011).

[12] Slater, J. C. & Koster, G. F. Simplified LCAO method for the periodic potential problem. *Phys. Rev.* **94**, 1498–1524 (1954).
